# Rubiscosome gene expression is balanced across the hexaploid wheat genome

**DOI:** 10.1007/s11120-022-00897-9

**Published:** 2022-01-27

**Authors:** Louis Caruana, Douglas J. Orr, Elizabete Carmo-Silva

**Affiliations:** Lancaster Environment Centre, Lancaster University, Lancaster, UK

**Keywords:** *Triticum aestivum*, Hexaploid wheat, Rubisco, Photosynthesis, Gene expression, Heat stress

## Abstract

Functional and active Rubisco is essential for CO_2_ fixation and is a primary target for engineering approaches to increasing crop yields. However, the assembly and maintenance of active Rubisco are dependent on the coordinated biosynthesis of at least 11 nuclear-encoded proteins, termed the ‘Rubiscosome’. Using publicly available gene expression data for wheat (*Triticum aestivum* L.), we show that the expression of Rubiscosome genes is balanced across the three closely related subgenomes that form the allohexaploid genome. Each subgenome contains a near complete set of homoeologous genes and contributes equally to overall expression, both under optimal and under heat stress conditions. The expression of the wheat thermo-tolerant Rubisco activase isoform 1β increases under heat stress and remains balanced across the subgenomes, albeit with a slight shift towards greater contribution from the D subgenome. The findings show that the gene copies in all three subgenomes need to be accounted for when designing strategies for crop improvement.

**Supplementary Information:**

The online version contains supplementary material available at 10.1007/s11120-022-00897-9.

## Introduction

The CO_2_ fixing enzyme of photosynthesis, ribulose-1,5-bisphosphate carboxylase/oxygenase (Rubisco), is a primary target for engineering efforts to increase the efficiency of photosynthesis in crops such as wheat. Rubisco biogenesis is complex and is further complicated by the hexaploid nature of the wheat genome. Here, we aim to address the research gap on the relative expression of Rubisco and its essential auxiliary factors across the multiple nuclear genomes of wheat. This information is essential in designing successful gene-editing approaches towards improving the agricultural productivity and climate resilience of wheat.

Plant Rubisco forms a hexadecamer, which is composed of eight large and eight small subunits. The large subunit is encoded by a single gene (rbcL) within the chloroplast genome, while the small subunit is encoded by a gene family (RbcS) located in the nuclear genome (Morita et al. 2016; Vitlin Gruber and Feiz 2018). Despite the spatial separation between the two genes, stoichiometry is maintained between the nuclear-encoded RbcS and the chloroplast-encoded rbcL at intermediate assembly stages. Mature RbcS upregulates the transcription of rbcL, while unassembled rbcL monomers downregulate the translation of further rbcL (Suzuki and Makino 2012; Wostrikoff and Stern 2007).

Plant rbcL monomers are highly prone to aggregation and cannot spontaneously fold into their functional form, requiring assistance from the nuclear-encoded chloroplast chaperonin complex (Bracher et al. 2017). The chloroplast chaperonin complex is predominantly composed of a tetradecamer of Cpn60 subunits arranged into two heptameric rings that form a cylindrical-like protein (Hayer-Hartl and Hartl 2020), and this is capped by a ring of Cpn10 and Cpn20 co-factors. Cpn20 is a tandem repeat of Cpn10 and is the most highly expressed chaperonin subunit in the chloroplast (Zhao and Liu 2018). Following binding of ATP, the chaperonin complex undergoes a conformational change, enclosing rbcL in a nano compartment that enables correct folding, the folded rbcL is subsequently released upon hydrolysis of the bound ATP (Bracher et al. 2017).

Rubisco holoenzyme (rbcL_8_RbcS_8_) assembly requires assistance from at least four known assembly chaperones, RbcX, Rubisco Accumulation Factor 1 (Raf1), Rubisco Accumulation Factor 2 (Raf2), and Bundle Sheath Defective 2 (BSD2) (Aigner et al. 2017). RbcX functions as a homodimer that binds specifically to the C-terminus of an rbcL peptide, prior to or following rbcL dimer formation, and disassociates from the rbcL_8_ core prior to binding of RbcS (Saschenbrecker et al. 2007). Raf1 associates with Rubisco assembly intermediates, binding to both RbcL_2_ and RbcL_8_ and has been proposed to facilitate the assembly of rbcL dimers (rbcL_2_Raf1_1_) into the octameric core (rbcL_8_Raf1_4_) (Hauser et al. 2015). Raf2 has been shown to interact with both rbcL and RbcS in the stroma (Feiz et al. 2014); its role remains unclear, but Raf2, like Raf1, appears to function as a post-chaperonin assembly chaperone (Aigner et al. 2017; Gruber and Feiz 2018). Bundle Sheath Defective 2 (BSD2) has also been suggested to operate as a post-chaperonin assembly chaperone (Feiz et al. 2014), stabilising the rbcL_8_ core in the absence of RbcS (Aigner et al. 2017; Vitlin Gruber and Feiz 2018; Conlan et al. 2019; Hayer-Hartl and Hartl 2020). The interactions of RbcX, Raf1, Raf2 and BSD2 with rbcL appear to be dynamic, and the four auxiliary factors seem to play somewhat overlapping roles, but all have been shown to be essential for in vitro Rubisco assembly (Aigner et al. 2017).

Following assembly of the holoenzyme, the active sites require post-translational modifications to become active. Carbamylation occurs when CO_2_ binds to a lysine (Lys-201) within the active site and is subsequently stabilised with the binding of a Mg^2+^ ion, rendering the enzyme catalytically competent, ready to bind the substrate ribulose-1,5-bisphosphate (Carmo-Silva et al. 2015). Following activation, naturally occurring sugar-phosphate derivative compounds can act as potent inhibitors. These inhibitory compounds, including 2-carboxy-D-arabinitol-1-phosphate (CA1P) and xylulose-1,5-bisphosphate (XuBP), play a key role in regulating Rubisco catalysis (Parry et al. 2008; Lobo et al. 2019). Inactive, inhibitor-bound Rubisco requires the function of its catalytic chaperone Rubisco activase (Rca), which releases the inhibitors from Rubisco in an ATP-dependant manner (Carmo-Silva et al. 2015). Following removal from Rubisco, the inhibitory compounds are subsequently degraded by the phosphatases CA1Pase and XuBPase (Sharwood 2017). rbcL, RbcS, Cpn60, Cpn20, RbcX, Raf1, Raf2, Rca, CA1Pase and XuBPase are all essential for Rubisco biogenesis and function and, therefore, can be collectively referred to as the ‘Rubiscosome’ (Erb and Zarzycki 2018).

Excluding rbcL, all other Rubiscosome proteins mentioned above are encoded by the nuclear genome. The nuclear genome of bread wheat contains a total of 21 chromosomes, consisting of the three distinct diploid genomes originating from the hybridisation of three closely related donor species. The first hybridization event occurred 300,000–500,000 years ago with the hybridisation of the diploid genome of *Triticum urartu* (AA) with the diploid genome of a closely related species to *Aegilops speltoides* (BB) forming the tetraploid *Triticum turgidum* (AABB) (Huang et al. 2002). The tetraploid genome of *T. turgidum* (AABB) was subsequently hybridised with the diploid genome of *Aegilops tauschii* (DD) forming the hexaploid genome of *T. aestivum* (AABBDD) around 10,000 years ago (Krasileva et al. 2013). Each donor genome (henceforth subgenome) contains a near identical set of homoeolog genes, forming homoeolog triads (IWGSC 2014). Genes previously subject to speciation (orthologous genes) become homoeologs when re-united in a single genome during allopolyploidization (Glover et al. 2016). Therefore, the expression of each of the nuclear-encoded Rubiscosome proteins in wheat originates from a homoeolog triad spanning the A, B and D subgenomes.

Despite homoeologs being on average 97.2% identical across coding sequences (Krasileva et al. 2017), variation exists within non-coding and repetitive sequences including intronic sequences of homoeolog genes, enabling the subgenome origin of transcripts to be determined (Feldman and Levy 2012). Balanced expression occurs when the total expression of a gene is derived from relatively equal quantities originating from each of the three homoeologs spanning the three subgenomes. In contrast, asymmetric gene expression occurs when most of the total expression of a gene is derived from just one or two of the three homoeologs. Analysis of triad expression of 53,259 wheat genes (Ramírez-González et al. 2018) showed that most triads were balanced (c.72.5%). The same study found that, within asymmetric triads characterised by varied contributions of the three subgenomes to the total expression of the respective gene, single subgenome suppression was more common (c.20.5%) than single subgenome dominance (c.7.1%). Overall expression of the D subgenome was slightly yet significantly more abundant than the B and A subgenomes (33.65%, 33.29% and 33.06%, respectively). As there is no recombination between chromosomes of the three genomes (Martinez-Perez et al. 2001), gene homoeologs that encode enzymes have a high degree of retention (Feldman et al., 2012). Therefore, multimeric enzymes such as Rubisco and the chaperonin complex are likely to feature subunits transcribed from homoeologs spanning all three wheat subgenomes. The aim of this study was to characterise the relative subgenome contributions to the expression of each Rubiscosome gene to inform biotechnological efforts aimed at improving Rubisco function in hexaploid wheat.

## Materials and methods

### Identification of Rubiscosome genes within the hexaploid wheat genome

In this study, ‘Rubiscosome’ genes include RbcS, Cpn60, Cpn20, Raf1, Raf2, Bsd2, RbcX, Rca1, Rca2, XuBPase and CA1Pase, with full names and functions listed in Table 1. rbcL is omitted due to being encoded on the chloroplast genome and, therefore, disparate from the hexaploid nuclear genome. The nuclear genome Rubiscosome genes were identified using the BLAST search feature on EnsemblPlants (Howe et al. 2020). Nucleic and amino acid sequences of Rubiscosome homologs from soybean (*Glycine max*), cowpea (*Vigna unguiculata*), maize (*Zea mays*), tobacco (*Nicotiana tabacum*) and Arabidopsis (*Arabidopsis thaliana*) were used for query sequences to assist in identifying wheat homologs (Feiz et al. 2012, 2014; Aigner et al. 2017; Lin et al. 2020).

**Table 1:** Names and functions of the Rubiscosome proteins explored in this study

Protein	Name	Function
BSD2	Bundle Sheath Defective 2	Rubisco Assembly Chaperone
CA1Pase	2-carboxy-D-arabinitol-1-phosphate Phosphatase	Catalytic Auxiliary Factor
Cpn20	Chaperonin 20	Chaperonin Subunit
Cpn60	Chaperonin 60	Chaperonin Subunit
Raf1	Rubisco Accumulation Factor 1	Rubisco Assembly Chaperone
Raf2	Rubisco Accumulation Factor 2	Rubisco Assembly Chaperone
Rca1/Rca2	Rubisco Activase	Rubisco Regulation
RbcS	Rubisco Small Subunit	Rubisco Subunit
RbcX	RbcX	Rubisco Assembly Chaperone
XuBPase	Xylulose-1,5-bisphosphate Phosphatase	Catalytic Auxiliary factor

Rubiscosome Gene_IDs that were identified from the BLAST analysis were collected and populated with relevant metadata including the encoded gene, gene locus coordinates, and all corresponding Transcript_IDs. Gene_IDs correspond to a gene locus within the wheat genome. A gene locus may contain several Transcript_IDs, each corresponding to a unique predicted transcript. Transcript_IDs are denoted by a decimal number at the terminus of a Gene_ID, for example, TraesCS4A02G177500.1 and TraesCS4A02G177500.2 are Transcript_IDs which correspond to the alpha and beta isoforms (respectively) of TraesCS4A02G177500, the A subgenome homoeolog locus of Rca2. To further ensure that the identified genes corresponded to the query genes, transcript and protein sequences for all Transcript_IDs were downloaded in FASTA format for comparative analysis to a homolog of a different species to the one used as the query sequence. Comparative analysis of transcript and peptide sequences were all performed using the Geneious Alignment feature of Geneious 9.1.8 (www.geneious.com).

### Rubiscosome Gene_IDs

Table 2 contains the Gene_IDs of all loci encoding Rubiscosome proteins. Gene_IDs were grouped together, by their subgenome location and by the Rubiscosome protein that they encode. The majority of the Rubiscosome proteins are encoded by an even number of loci which have been mapped to the A, B and D subgenomes, with some exceptions, detailed below.

**Table 2:** Gene identifiers for known components of the Rubiscosome in wheat

Gene	A Subgenome	B Subgenome	D Subgenome
*Bsd2*	TraesCS7A02G341000	TraesCS7B02G242200	TraesCS7D02G338600
*CA1Pase*	TraesCS4A02G184100	TraesCS4B02G134600	TraesCS4D02G129300
*Cpn20*	TraesCS6A02G340300 TraesCS5A02G212500 TraesCS7A02G161000 TraesCS2A02G146000	TraesCS6B02G371500 TraesCS5B02G211200 TraesCS7B02G066000 TraesCS2B02G171400	TraesCS6D02G320800 TraesCS5D02G219500 TraesCS7D02G162300 TraesCS2D02G150600
*Cpn60*	TraesCS4A02G315500 TraesCS5A02G366800	TraesCS5B02G563900 TraesCS5B02G368900	TraesCS5D02G550700 TraesCS5D02G376000
*Raf1*	TraesCS1A02G142000	TraesCS1B02G159700	TraesCS1D02G141100
*Raf2*	TraesCS5A02G545700	TraesCS4B02G379500	TraesCSU02G129700
*RbcS*	TraesCS2A02G066700 TraesCS2A02G066800 TraesCS2A02G066900 TraesCS2A02G067000 TraesCS2A02G067100 TraesCS2A02G067200 TraesCS2A02G067300 TraesCS5A02G165400 TraesCS5A02G165700	TraesCS2B02G079100 TraesCS2B02G079200 TraesCS2B02G079300 TraesCS2B02G079400 TraesCS2B02G079500 TraesCS2B02G078900 TraesCS5B02G162600 TraesCS5B02G162800	TraesCS2D02G065100 TraesCS2D02G065200 TraesCS2D02G065300 TraesCS2D02G065400 TraesCS2D02G065500 TraesCS2D02G065600 TraesCS5D02G169600 TraesCS5D02G169900
*RbcX*	TraesCS2A02G198700 TraesCS5A02G459200	TraesCS2B02G226100 TraesCS5B02G468800	TraesCS2D02G206500 TraesCS5D02G470300
*Rca1*	TraesCS4A02G177600	TraesCS4B02G140200	TraesCS4D02G134900
*Rca2*	TraesCS4A02G177500	TraesCS4B02G140300	TraesCS4D02G135000
*XuBPase*	TraesCS7A02G335600	TraesCS7B02G247200	TraesCS7D02G343300

Nomenclature of the A subgenome homoeolog of *Bsd2* Gene ID explained: ‘Traes’ refers to the species Triticum aestivum; CS refers to the accession, Chinese Spring; 7A refers to chromosome 7, subgenome A; 02 refers to RefSeq v1.1; G refers to the locus encoding a Gene; 341,000 is the unique identifier for this locus

The *Raf2* A (TraesCS5A02G545700) and B (TraesCS4B02G379500) homoeologs have been mapped to chromosomes successfully in the reference genome used in this study. A blast search query of the A and B sequences also returned a Gene_ID (TraesCSU02G129700) which had been mapped to an unassigned chromosome category in the reference genome. A sequence alignment of the mature protein sequence of these three Gene_IDs returned a 95.9% pairwise identity. Therefore, the unassigned TraesCSU02G129700 was assumed to be the D subgenome homoeolog of *Raf2*.

The *RbcS* loci identified are not balanced equally in number across the three subgenomes with the A, B and D subgenomes containing 9, 8 and 8 homoeologs, respectively. It is not possible to determine which of the loci are homoeologous. For the purpose of these analyses, expression data from each of *RbcS* loci have been grouped by subgenome, meaning that the results for *RbcS* represent the total gene expression conferred by the gene copies across the respective subgenomes rather than per homoeolog.

### Expression data collection

The wheat-expression browser (www.wheat-expression.com) contains expression data from 36 independent studies (as of July 2021), incorporating a broad range of biotic and abiotic stress conditions (Borrill et al. 2016). To establish the expression of Rubiscosome genes under reasonably consistent and stable conditions, and to prevent the results being influenced by any stress imposed on the plants, six studies were selected which stated similar photoperiod and temperature regimes for their plant growth conditions (Table 3). To allow for the inclusion of more datasets and increase the robustness of the analysis, studies were selected, which included samples taken at varied growth stages, with the intention to characterise Rubiscosome expression broadly across green tissues during wheat growth.

**Table 3:** Reported photoperiod, temperature regime, variety, and growth stage in seven studies selected from the wheat-expression browser (www.wheat-expression.com)

Study	Day:Night Length (h)	Day:Night Temperature (°C)	Heat Stress Temperature (°C)	Wheat Variety	Growth Stage	Study Number
Developmental time course of Chinese Spring (Ramírez-González et al., 2018)	16:8	25:15	NA	Chinese Spring	Seedling, Seven Leaf, Tillering, Anthesis, 2 Days Post Anthesis, Two Nodes Detectable	1
Chinese Spring seedling and spikes at anthesis (Ramírez-González et al., 2018)	12:12	20	NA	Chinese Spring	14 Days Old, Anthesis	2
Chinese Spring leaves and roots from seven leaf stage (Ramírez-González et al., 2018)	12:12	20	NA	Chinese Spring	Seven Leaf	3
Chinese Spring early meiosis, early prophase (Martín et al., 2018)	16:8	20:15	NA	Chinese Spring	Early Booting (39 Zadoks)	4
Developmental time course of Azhurnaya (Ramírez-González et al., 2018)	16:8	25:15	NA	Azhurnaya	Seedling, Three Leaf, Fifth Leaf, Tillering, Flag Leaf, Full Boot, 30% spike, Ear emergence, Anthesis, Milk Grain, Dough	5
Gene expression during a time course of flag leaf senescence (Borrill et al., 2019)	16:8	20:15	NA	Bobwhite	3, 7, 10, 13, 15, 17, 19, 21, 23, 26, Days after anthesis	6
*Drought and heat stress time course in seedlings (Liu et al., 2015)	16:8	22:18	40	TAM107	Seedling	7

*Data from Liu et al., (2015) was exclusively used for heat stress analysis

Reference assemblies have struggled to compile the full hexaploid genome due to its large size (~ 16 Gb) and repetitive sequences (~ 85%). The gene coordinates and annotations of the 2018 RefSeq1.1 assembly were utilised in this study since this has successfully mapped 14.1 Gb of the wheat genome to the 21 chromosomes and a further 481 Mb to an ‘unassigned chromosome’ (IWGSC 2018).

### Expression data analysis and visualisation

Sample specific expression data per Gene_ID (Table 2) were downloaded from the wheat-expression browser in transcripts per million (tpm) format. The mean of the samples per Gene_ID was then calculated. In the case of proteins that were encoded by multiple loci per sub genome, the mean tpm per Gene_ID was summed to give total tpm per gene per subgenome:

$$\begin{aligned}&Total\, A\, subgenome \,expression \,of \,Cpn60\,=\\&\,TraesCS4A02G315500 \,tpm+TraesCS5A02G366800 \,tpm.\end{aligned}$$

In order to ensure that the relative expression of each of the Rubiscosome proteins was standardised across subgenomes, the relative expression per subgenome of each protein was expressed as a fraction of the total:

$$\begin{aligned}&Relative \,A\, subgenome \,expression=\\&\frac{\left(Total \,A\, tpm\right)}{\left(Total \,A\, tpm\right)+\left(Total \,B \,tpm\right)+(Total \,D \,tpm)},\end{aligned}$$

$$\begin{aligned}Relative \,B \,subgenome \,expression=\\&\frac{\left(Total \,B \,tpm \right)}{\left(Total \,A\, tpm\right)+\left(Total \,B\, tpm\right)+ (Total \,D\, tpm)},\end{aligned}$$

$$\begin{aligned}Relative \,D\, subgenome \,expression=\\&\frac{\left(Total \,D \,tpm\right)}{\left(Total \,A \,tpm\right)+\left(Total \,B \,tpm\right)+(Total \,D\, tpm)}.\end{aligned}$$

Finally, to visualise the total expression per Rubiscosome protein, the sum of total tpm per subgenome was calculated and Log2 transformed:

$$\log 2\left(\left(Total \,A\, tpm\right)+\left(Total \,B \,tpm\right)+\left(Total \,D \,tpm\right)\right).$$

**Fig. 1:**
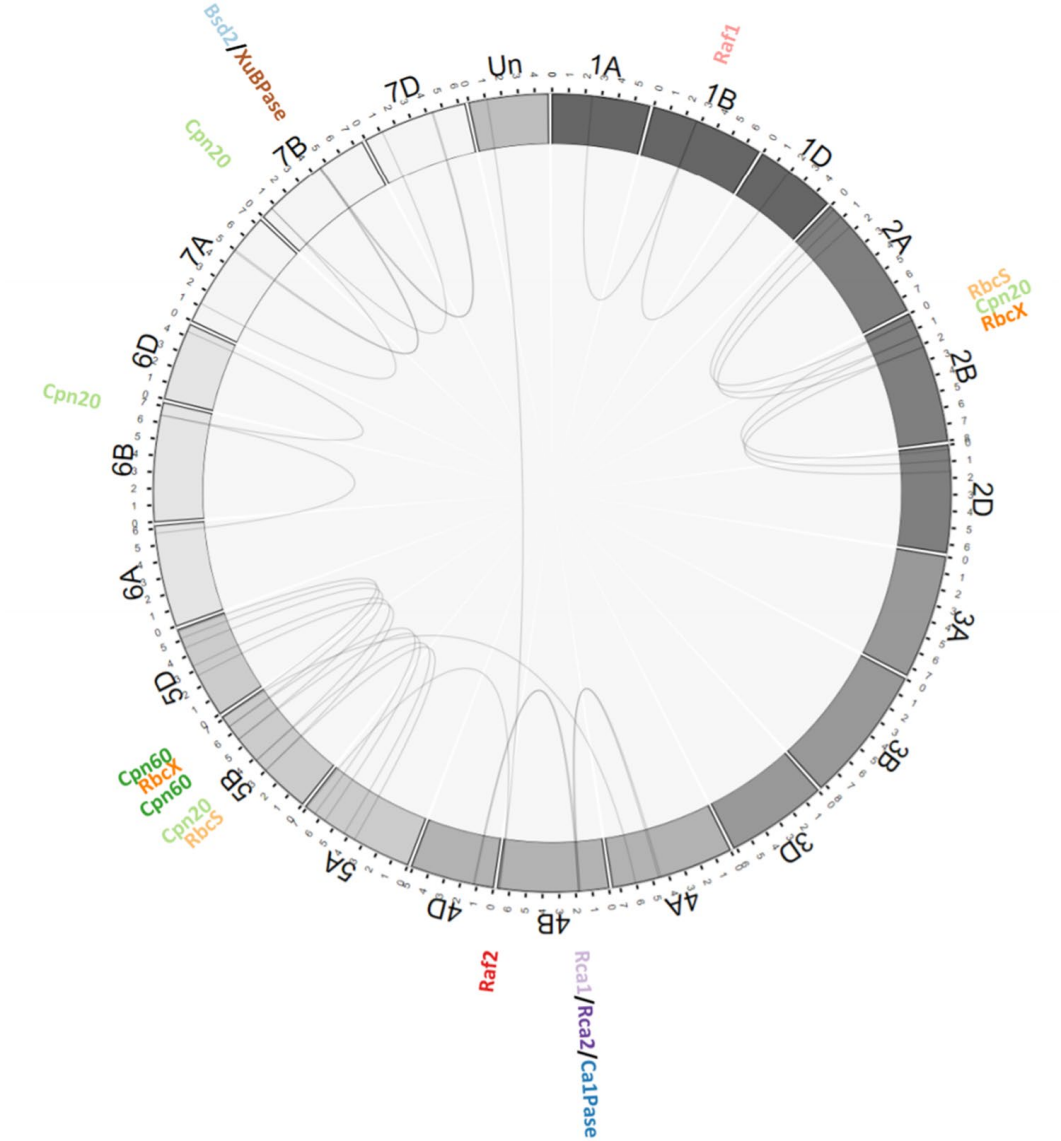
Circular visualisation of the hexaploid wheat genome and the position of the homoeolog triads used in this study. The tracks from the outside to the centre specify: names of each homoeolog triad; chromosome name and length (100 Mb tick size). Connecting lines represent homoeologous relationships between genes across chromosomes in subgenomes. Chromosome ‘Un’ indicates homoeologs unallocated to a chromosome position, i.e. within the ‘unassigned chromosome’ of the RefSeq1.1 reference genome

**Fig. 2:**
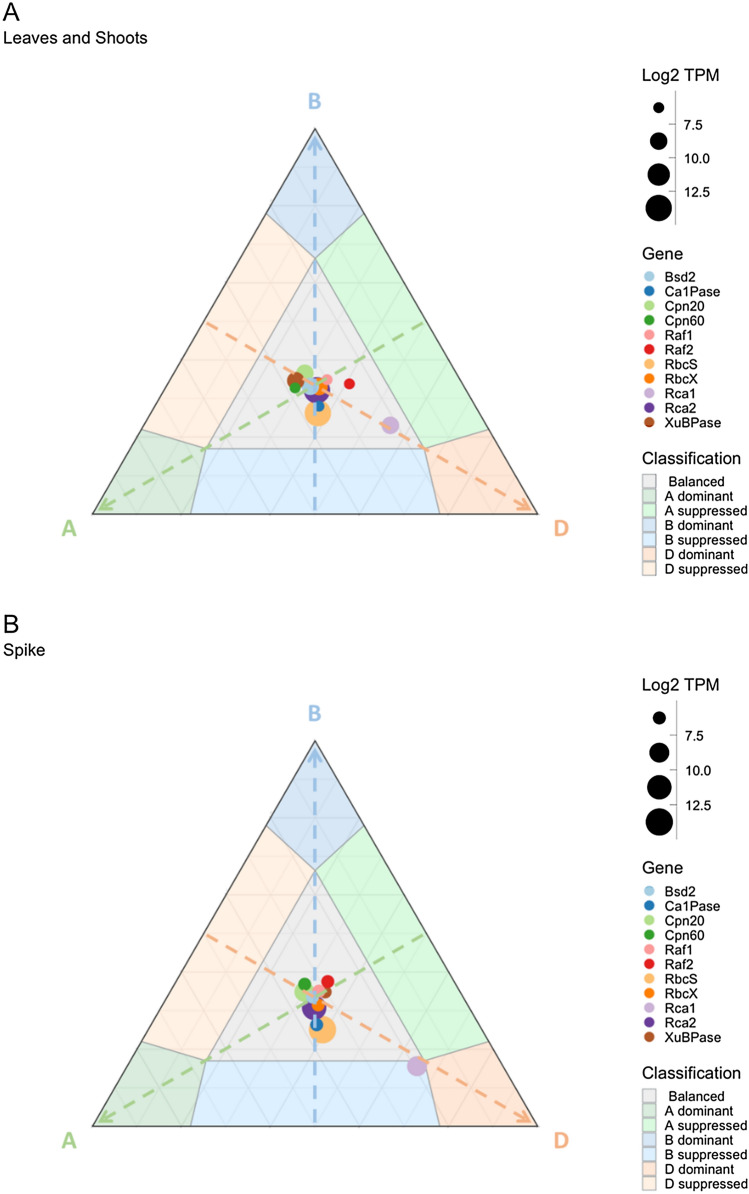
Relative expression and expression balance of Rubiscosome triads in the **A** leaves and shoots and **B** spike of hexaploid wheat from six comparable studies (Table 3). The three arrows each represent increasing expression of a subgenome indicated by the letter. The position of each symbol represents the relative contribution of each subgenome-specific homoeolog to the overall expression of the respective gene. The size of each symbol is representative of the total expression of each gene triad (Log2 TPM)

**Fig. 3:**
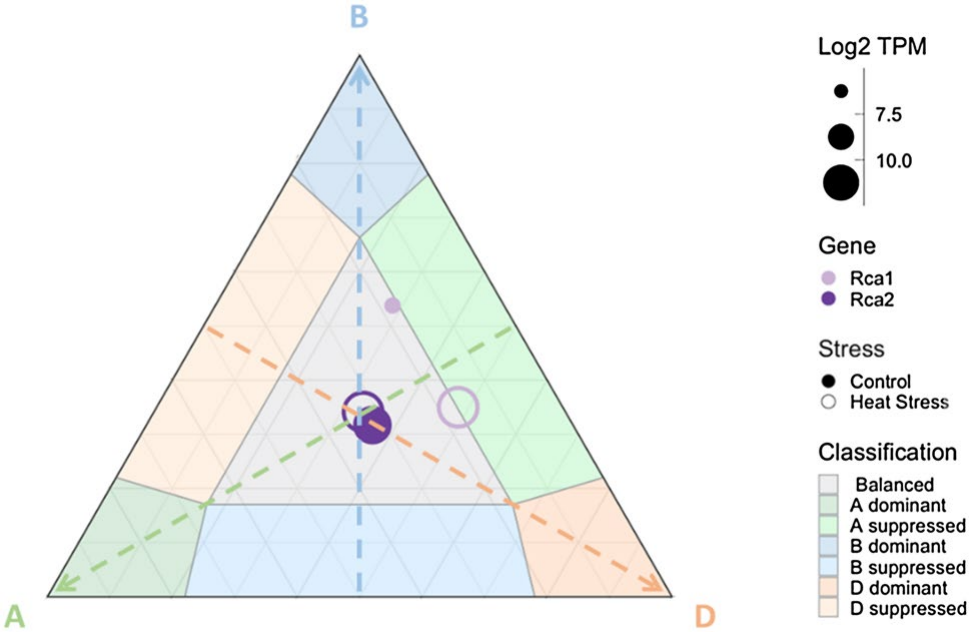
Relative expression and expression balance of Rca1 and Rca2 in leaves and shoots of hexaploid wheat heat tolerant cultivar TAM107 under control and heat stress conditions. The three arrows each represent increasing expression of a subgenome indicated by the letter. The position of each symbol represents the relative contribution of each subgenome-specific homoeolog to the overall expression of the gene. The size of each symbol is representative of the total expression of each gene triad (Log2 TPM). Data from Liu et al. (2015) were used for heat stress analysis

All data wrangling was completed using the R Language, tidyr and dplyr packages as part of the Tidyverse (Wickham et al. 2019). Figure 1 is generated using the R language adaption of BioCircos.js (Cui et al. 2016). Ternary diagrams (Figs. 2–3) were generated using ggtern package (Hamilton and Ferry 2018). Code of the analysis is available at https://github.com/LouisCaruana/Wheat-Rubiscosome-Expression-Balance-

## Results

### Identification of Rubiscosome homoeolog loci within the hexaploid wheat genome

The blast search of the wheat hexaploid nuclear genome returned a total of 70 gene loci that encoded Rubiscosome proteins. The Rubiscosome genes were well distributed across the wheat chromosomes (Fig. 1), with only the chromosome 3 triplicate not encoding any Rubiscosome genes. Most of the Rubiscosome genes showed a 1:1:1 correspondence of homoeologs across the three subgenomes. However, this was not true of the multiple *RbcS* gene loci, which located mostly in tandem in chromosomes 2 and 5. In chromosome 2, there were six *RbcS* copies per subgenome plus an additional copy on chromosome 2A, and in chromosome 5, there were two *RbcS* copies per subgenome.

The chromosomal positions of each Rubiscosome gene triad are visualised by the connecting lines in Fig. 1. With a few exceptions, the A, B and D homoeologs of each gene triad tended to show a similar position on the respective chromosomes. Cpn20 was encoded by four discrete gene triads (Cpn20_1 to Cpn20_4), on chromosomes 2, 4, 6 and 7. Of the two discrete Cpn60 gene triads, Cpn60_2 was encoded on chromosome 5 across the three subgenomes, while Cpn60_1 homoeologs have been mapped to chromosomes 4A, 5B and 5D. The assumed D subgenome homoeolog of Raf2 has not been mapped to a chromosome in the reference genome used and, therefore, is displayed in the unassigned chromosome. The other two subgenome homoeologs of Raf2 have been mapped to chromosomes 5A and 4B. The homoeolog loci of these gene triads spanning separate chromosome triplicates are consistent with known translocation events within the wheat genome (IWGSC 2018).

### Relative subgenome expression of the Rubiscosome is consistent across tissue types

The A, B and the D loci of the majority of the Rubiscosome genes contributed equally to the total gene expression of their respective genes in the studies used. Bsd2, CA1Pase, Cpn20, Cpn60, Raf1, Raf2, RbcS, RbcX, Rca2 and XuBPase genes were all expressed similarly by their respective loci in the leaves and shoots of hexaploid wheat, as shown by the cluster of points in the centre of a ternary plot of expression balance (Fig. 2A). The expression data available also allowed for an assessment of homoeolog expression balance in wheat spike tissue (Fig. 2B). Consistent with the largely balanced expression seen in shoot and leaf tissues, wheat spikes were also observed to have balanced expression for the Rubiscosome genes, except for Rca1. The two ternary plots display a nearly identical data spread with most of the points clustering in the centre of the plots, indicative of balanced expression among the 3 subgenomes in both tissue types. This suggests a constitutive mechanism underpinning tissue-independent relative gene expression by each respective locus.

Expression of Rca1 stood out as relatively asymmetric when compared to the other Rubiscosome genes. Total Rca1 expression in the leaves and shoots of hexaploid wheat was composed of 22%, 23% and 55% from the A, B and D subgenomes, respectively; however, it still fell within what is generally considered balanced expression. The trend towards asymmetric expression of Rca1 was more pronounced in the spike tissues, composed of 19%, 16% and 65% from the A, B and D subgenomes, respectively. Rca1 expression in the spike fell on the boundary of balanced expression, A and B subgenome suppression and D subgenome dominance.

### Heat stress alters the relative subgenome expression of some, not all, Rubiscosome genes

To assess how the expression balance of Rubiscosome gene homoeologs may be impacted by heat stress, an important abiotic stressor of wheat photosynthesis, the same analysis was carried out on samples from a heat tolerant wheat variety, TAM107 (Liu et al. 2015). Based on this analysis, Rubiscosome gene expression could be broadly split into two groups based on whether there were dynamic changes under heat stress compared to control conditions. Bsd2, CA1Pase, Cpn20, Raf2, RbcS, Rca2 and XuBPase showed no change, while Cpn60, Raf1, RbcX and Rca1 all displayed changes in their expression balance in response to heat stress (Fig. S1).

Cpn60 shifted from balanced expression across subgenomes under control conditions to B subgenome supressed under heat stress. Raf1 expression shifted towards D subgenome suppression but remained within the balanced expression category. RbcX expression shifted towards A subgenome suppression in response to heat stress and displayed a considerable upregulation in total expression. Rca1 displayed the largest shift in expression balance. Under control conditions, greater than half of Rca1 expression is from the B subgenome homoeolog (17%, 53% and 28% from the A, B and D subgenomes, respectively, Fig. 3). However, under heat stress conditions, Rca1 expression becomes more evenly split between the B and D subgenomes, while the contribution of the A subgenome remains low and near classification as A subgenome supressed. Rca1 total expression also massively increased from 102 transcripts per million under control conditions to 3152 transcripts per million when subjected to heat stress conditions.

The increased expression of Rca1 subgenome D homoeolog under heat stress raised the question of whether the proteins encoded by the three homeologs would differ in sequence. While sequences of TAM107 Rca1 homoeologs were not available to us, comparison of the coding sequences (Fig. S2) and mature protein sequences (Fig. S3) of wheat Rca1 (*Triticum aestivum* cv. Chinese Spring; IWGSC 2018) shows high homology, with 96.8% identical coding sequences and 99% identical protein sequences. The mature Rca1 protein sequences corresponding to the A and D subgenome homeologs are identical, with the protein resulting from the B subgenome homoeolog featuring 4 amino acid polymorphisms (Table S1).

## Discussion

Rubisco, the primary carbon-fixing enzyme, can constitute up to 50% of total protein in leaves of C3 plants such as wheat (Parry et al. 2003; Carmo-Silva et al. 2015) and is a prime target for improving the efficiency of photosynthesis. Leaves are the primary photosynthetic organs of wheat; however, the importance of photosynthesis in non-foliar tissues is increasingly recognised, with spike tissues shown to contribute up to 39% of grain biomass (Zhang et al. 2020). Given the hexaploid genome of wheat, we set out to characterise the relative subgenome contribution to the expression of known nuclear-encoded genes related to the synthesis and function of Rubisco, termed the Rubiscosome. This analysis used publicly available data for gene expression in leaf and spike tissues of hexaploid wheat (Borrill et al. 2016). The findings will inform approaches for improving Rubisco biogenesis, activity and regulation aimed at enhancing agricultural crop productivity.

A total of seventy gene loci were identified across the wheat genome which encode proteins currently known to be essential for Rubisco biogenesis and function. Due to the similarity of the three subgenomes, the three homoeologs corresponding to each gene triad were generally found to occur in a similar location on the respective chromosomes. However, homoeologs of Raf2 and Cpn60 are located within translocated regions (Clavijo et al. 2017), resulting in gene triads that span multiple chromosomes. The chaperonin Cpn20 was encoded by four distinct gene triads spread across four separate chromosomes, and the RbcS gene family was composed of tandemly organised genes in chromosomes 2 and 5. The redundancy of RbcS gene copies might be explained as either a gene function protective mechanism, or a subfunctionalisation mechanism, in the ancestral species of the three diploid progenitors (Yamada et al. 2019).

The Rubiscosome gene expression was generally well balanced across the three subgenomes in the leaves and spike tissues of hexaploid wheat (Fig. 2), i.e. there was no clear dominant subgenome contribution towards overall Rubiscosome expression. This is consistent with previous reports that the expression of over 70% of homoeolog triads are balanced (Ramírez-González et al. 2018). The total expression conferred by the gene triads was also consistent between the leaves and spike tissues, suggesting that a functional Rubiscosome is essential for both leaf and spike photosynthesis.

The gene loci encoding Rca1 did not display the same balanced expression as observed for the majority of Rubiscosome genes. Instead, Rca1 featured varying degrees of asymmetric expression. A previous report stated that, based on unpublished expressed sequence tags (EST) data, Rca1 and Rca2 were most highly expressed by the B subgenome (Carmo-Silva et al. 2015). The results reported herein, based on the gene expression data from 7 studies, disagree with this affirmation. Rca2 expression remained consistently balanced across the three subgenomes in different wheat cultivars, plant tissues and under heat stress conditions (Figs. 2 and 3). Rca1 expression displayed a more dynamic pattern, with a trend towards subgenome D dominance in leaves and spikes (Fig. 2).

Analysis of expression data for the heat tolerant wheat variety TAM107 (Liu et al. 2015) showed an increase in Rca1 expression in seedlings exposed to heat stress (40 °C) for up to 6 h relative to control temperatures of 18–22 °C (Fig. 3). This observation is consistent with the 40-fold increase in Rca1 gene expression reported for wheat plants after 4 h exposure to heat stress (38 °C), with no corresponding increase in Rca2 expression (Degen et al. 2021). The wheat Rca1 gene triad encodes a short isoform of Rca, while the Rca2 gene triad produces both a short and a longer isoform via alternative splicing (Carmo-Silva et al., 2015). Rca1 protein has been shown to feature greater thermostability than the two Rca2 isoforms (Scafaro et al. 2019; Degen et al. 2020). Upregulation of Rca1 expression in wheat plants under heat stress has been attributed to a heat responsive element that is present in the promotor regions of all three Rca1 homoeologs, while this is only present in the A homoeolog of Rca2 (Jung et al. 2013; Degen et al. 2021).

The relative expression asymmetry of Rca1 in TAM107 wheat appears to be dynamic. While expression was upregulated by all three loci under heat stress conditions relative to control, the D subgenome displayed a much greater increase than the A and the B subgenome. This resulted in a shift in expression towards D subgenome dominance, although overall expression remained balanced across the subgenomes (Fig. 3). Increased expression of other gene homeologs from subgenome D has been previously noted under abiotic stress (Liu et al., 2015). While in Chinese Spring wheat, the Rca1 protein encoded by subgenomes A and D is identical and there are only 4 amino acid residue differences relative to the Rca1 protein encoded by subgenome B (Fig. S3), TAM107 may contain further polymorphisms in Rca1 among subgenomes. Similarly, it is possible that the presence of heat responsive elements might show genotypic variation and the potential role of heat responsive elements in Rca gene expression is an area that warrants further investigation as a possible target for manipulating Rubisco regulation under heat stress.

In conclusion, these results demonstrate that Rubiscosome genes are expressed in a balanced manner across the three wheat subgenomes, and this balanced expression is consistent across plant tissues. The findings resolve some uncertainty on the contribution of the three subgenomes to the expression of the key photosynthetic regulatory protein Rca in hexaploid wheat. Except for the relatively asymmetric expression observed in Rca1, there was no dominant subgenome contribution towards overall expression of the remaining Rubiscosome proteins. Therefore, gene-editing strategies aiming to increase CO_2_ fixation by targeting Rubiscosome components should ensure that all the target homoeologs are successfully edited to ensure consistent changes in gene expression and resulting phenotype.

## Supplementary Information

Below is the link to the electronic supplementary material.

Supplementary file1 (DOCX 406 kb)

## Data Availability

Gene expression data were available from the wheat-expression Browser at www.wheat-expression.com

## Code availability

The code used in the analysis is available at https://github.com/LouisCaruana/Wheat-Rubiscosome-Expression-Balance-
