# Supplementary material for: Rubiscosome gene expression is balanced across the hexaploid wheat genome
Source: Photosynth Res. 2022 Jan 27;152(1):1–11. doi: 10.1007/s11120-022-00897-9 (PMC9090852; doi:10.1007/s11120-022-00897-9)
Supplement: Supplementary file 1 — Supplementary file1 (DOCX 406 kb) [file 11120_2022_897_MOESM1_ESM.docx]

**Rubiscosome gene expression is balanced across the hexaploid wheat genome**

Louis Caruana, Douglas J. Orr, Elizabete Carmo-Silva*

Lancaster Environment Centre, Lancaster University, Lancaster, UK

* For correspondence (Email: [e.carmosilva@lancaster.ac.uk](mailto:e.carmosilva@lancaster.ac.uk))


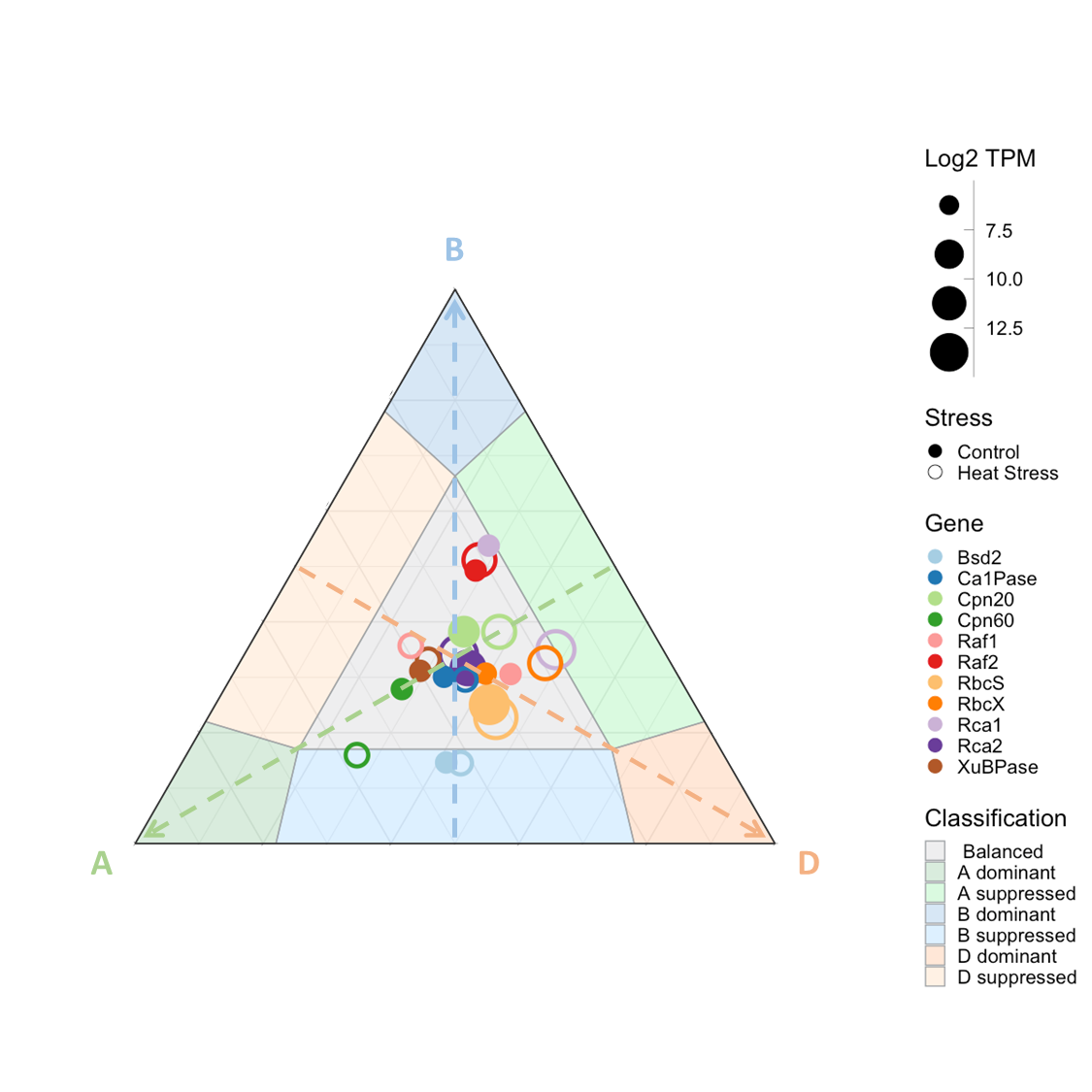


**Figure S1** Relative expression and expression balance of Rubiscosome genes in leaves and shoots of hexaploid wheat heat tolerant cultivar TAM107 under control and heat stress conditions. The three axes each correspond to a subgenome indicated by the letter. The position of each symbol represents the relative contribution of each subgenome specific homoeolog to the overall expression of its respective gene. The size of each symbol is representative of the total expression of each gene triad (Log2 TPM). Data from Liu *et al*. (2015) was used for heat stress analysis.


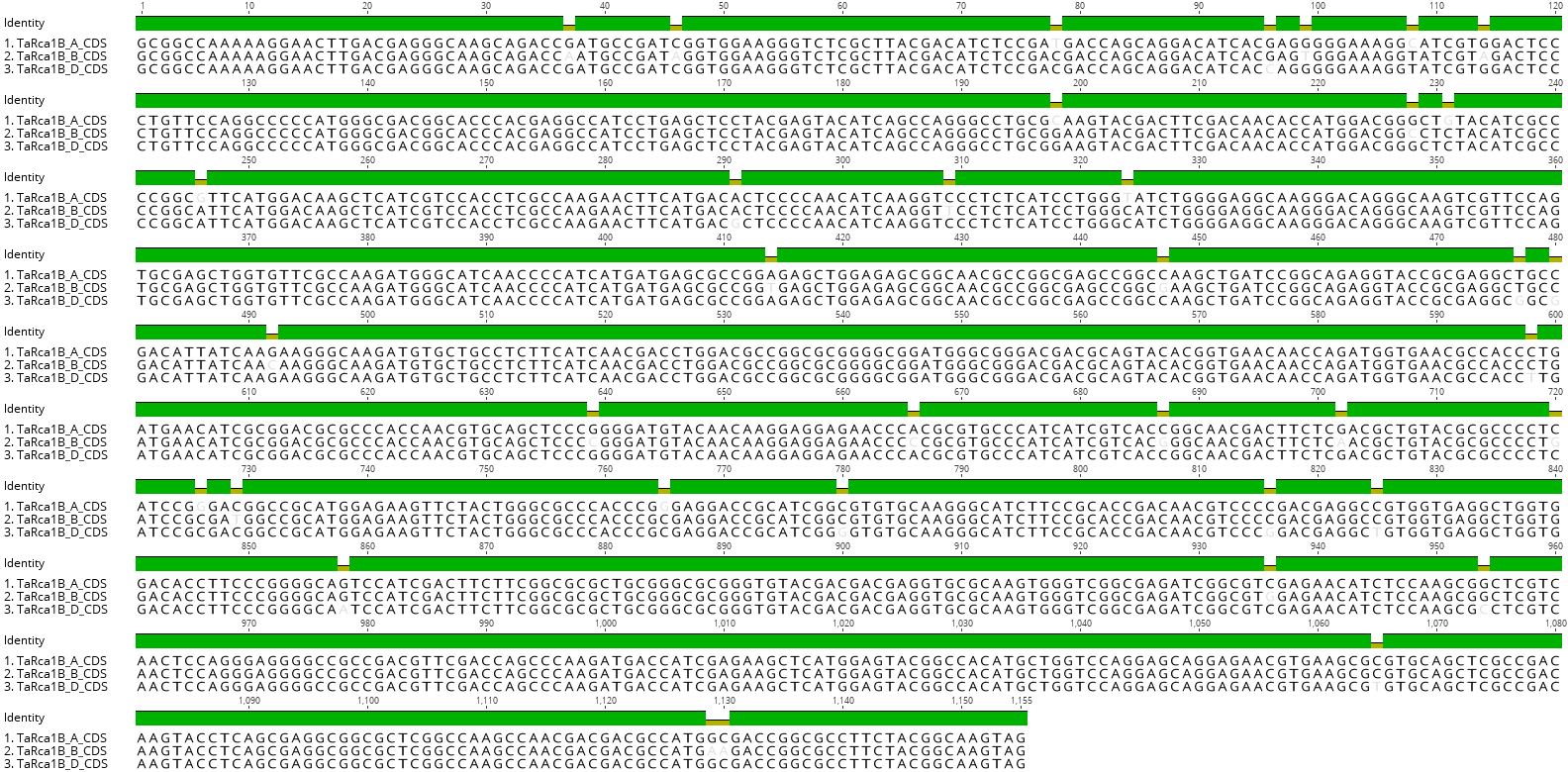


**Figure S2** Coding sequence alignment of the wheat A, B, and D homoeologs for mature Rca1 protein (cv. Chinese Spring, IWGSC 2018). The green and olive bar indicates consensus; green shows regions of identical sequence consensus, olive highlights nucleotide differences between *Rca1* homoeologs. Overall, the three sequences feature 96.8% identical sites, with 8, 17, and 11 nucleotide polymorphisms in the A, B, and D homoeologs respectively, as compared with the consensus.

**
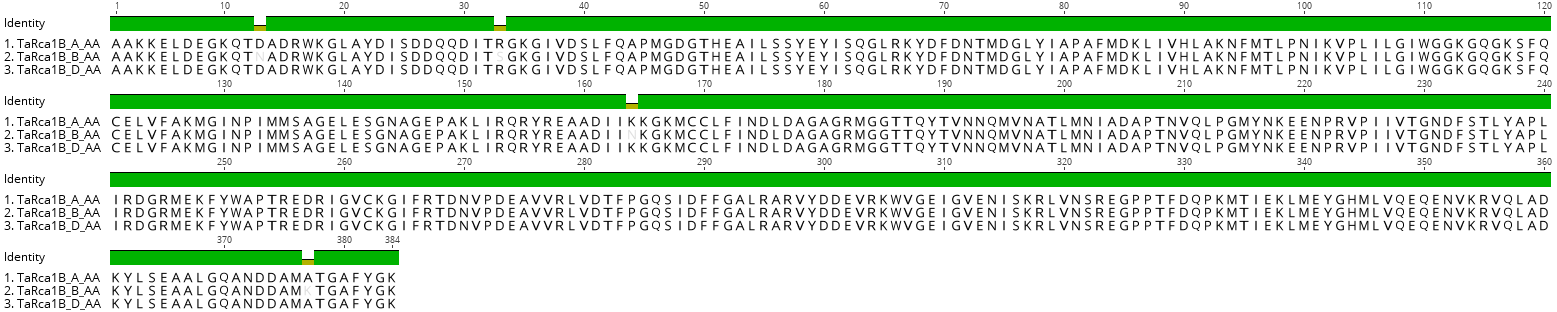
**

**Figure S3** Protein sequence alignments of the wheat A, B, and D homoeologs of mature Rca1, post transit peptide cleavage. The green and olive bar indicates consensus; green shows regions of consensus, olive highlights amino acid polymorphisms. Overall, the three sequences feature 99% identical amino acid residues; the protein resulting from expression of the B subgenome homoeolog has 4 amino acid polymorphisms, the sequences encoded by the A and D subgenomes are identical.

**Table S1** Summary of the comparison of mature wheat Rca1 coding sequences and amino acid sequences from the A, B, and D subgenomes in hexaploid wheat.

| **Subgenome** | **Mature sequence length** | **Nucleotide / amino acid polymorphisms** | **% Identical sites** |
| --- | --- | --- | --- |
| *Coding Sequence* | | | |
| A | 1155 | 8 | 96.8% |
| B | 1155 | 17 |  |
| D | 1155 | 11 |  |
| *Amino Acid Sequence* | | | |
| A | 384 | 0 | 99% |
| B | 384 | 4 |  |
| D | 384 | 0 |  |
